# Analysis of the complete genome sequence of *Paenibacillus* sp. lzh-N1 reveals its antagonistic ability

**DOI:** 10.1186/s12864-024-10206-4

**Published:** 2024-03-13

**Authors:** Ee Li, Kaiquan Liu, Shuhan Yang, Ling Li, Kun Ran, Xiaoli Sun, Jie Qu, Li Zhao, Yuxiu Xin, Feng Zhu, Jingfang Ma, Feng Song, Zhenghua Li

**Affiliations:** 1 Shandong Key Laboratory of Biophysics, Institute of Biophysics, Dezhou University, 253023 Dezhou, P. R. China; 2 Shandong Engineering Laboratory of Swine Herd Health Big Data and Intelligent Monitoring, Dezhou University, 253023 Dezhou, P.R. China; 3 School of Bioengineering, Qilu University of Technology, Shandong Academy of Sciences, 250353 Jinan, P. R. China; 4 Shandong Institute of Pomology, 271000 Taian, P. R. China

**Keywords:** *Paenibacillus* sp., Genome sequence, Antagonistic ability, Antifungal peptides, Biofilm formation, Quorum sensing

## Abstract

**Background:**

Plant diseases caused by pathogenic fungi are devastating. However, commonly used fungicides are harmful to the environment, and some are becoming ineffective due to fungal resistance. Therefore, eco-friendly biological methods to control pathogenic fungi are urgently needed.

**Results:**

In this study, a strain, *Paenibacillus* sp. lzh-N1, that could inhibit the growth of the pathogenic fungus *Mycosphaerella sentina* (Fr) Schrorter was isolated from the rhizosphere soil of pear trees, and the complete genome sequence of the strain was obtained, annotated, and analyzed to reveal the genetic foundation of its antagonistic ability. The entire genome of this strain contained a circular chromosome of 5,641,488 bp with a GC content of 45.50%. The results of species identification show that the strain belongs to the same species as *P. polymyxa* Sb3-1 and *P. polymyxa* CJX518. Sixteen secondary metabolic biosynthetic gene clusters were predicted by antiSMASH, including those of the antifungal peptides fusaricidin B and paenilarvins. In addition, biofilm formation-related genes containing two potential gene clusters for cyclic lactone autoinducer, a gene encoding S-ribosylhomocysteine lyase (LuxS), and three genes encoding exopolysaccharide biosynthesis protein were identified.

**Conclusions:**

Antifungal peptides and glucanase biosynthesized by *Paenibacillus* sp. lzh-N1 may be responsible for its antagonistic effect. Moreover, quorum sensing systems may influence the biocontrol activity of this strain directly or indirectly.

## Background

Plant diseases caused by pathogenic fungi are devastating, which may lead to severe crop losses. A variety of diseases caused by fungi have been controlled by chemical fungicides. However, most of these fungicides are harmful to the environment, and some are not effective in eradicating pathogens as many pathogens have become resistant to antifungal drugs [1]. Therefore, biological methods to protect crops from plant diseases have received considerable attention. Plant-growth-promoting rhizobacteria (PGPR), a group of rhizosphere bacteria, can improve plant growth through various mechanisms, such as biological nitrogen fixation, siderophore production, antibiotic secretion, and systemic resistant induction [2], making them the most promising biocontrol agents to control diseases affecting crop production.

Among PGPR, bacteria in the genus *Paenibacillus* (e.g., *P. polymyxa*) have attracted global interest as they have native beneficial properties to apply in the modern agriculture. *Paenibacillus* sp. can promote plant growth by improving nitrogen fixation, iron acquisition, and phosphorus solubilization [3]. More importantly, *Paenibacillus* sp. can secrete diverse beneficial bioactive metabolites including antimicrobial polypeptides, volatile organic compounds, and hydrolytic enzymes, which can reduce the reliance on chemical antimicrobial agents. Furthermore, *Paenibacillus* sp. has shown extensive environmental adaptability [4–6]. These characters enable the bacteria to be widely used in the biofertilizer for improving plant growth and for maintaining a sustainable agroecosystem simultaneously.

*Paenibacillus* sp. lzh-N1 was isolated from the rhizosphere of pear in Taian, Shandong Province of China. It has excellent inhibitory effects on the pathogen *Mycosphaerella sentina* (Fr) Schrorter. In the present study, the complete genome sequence of *Paenibacillus* sp. lzh-N1 was obtained, and the corresponding genes were annotated. In addition, the secondary metabolite biosynthetic gene clusters (BGCs), genes encoding hydrolytic enzymes, and potential quorum sensing (QS) systems were analyzed to elucidate the genetic background and molecular mechanism underlying the antagonistic ability of this strain.

## Results

### The biocontrol efficacy of *Paenibacillus* sp. lzh-N1

**Fig. 1:**
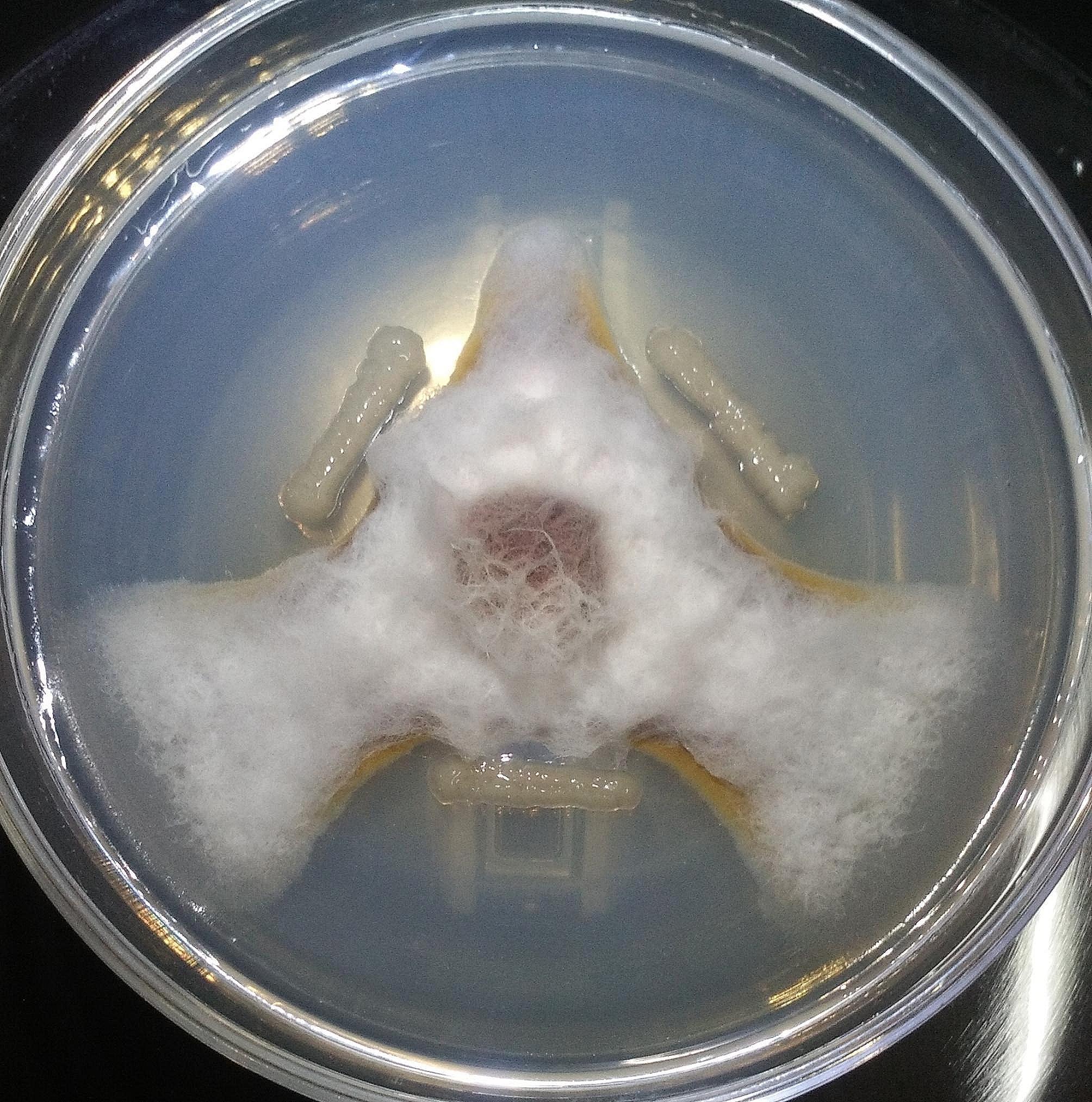
In vitro antagonistic activities of *Paenibacillus* sp. lzh-N1 against *M. sentina* (Fr) Schrorter

The antagonistic activity of *Paenibacillus* sp. lzh-N1 against *M. sentina* (Fr) Schrorter, a destructive pathogenic fungus for pear in China, was evaluated. *Paenibacillus* sp. lzh-N1 showed excellent antagonistic activity against the pathogen (Fig. 1), indicating that this strain is promising to improve pear growth as a biocontrol agent. *Paenibacillus* sp. lzh-N1 has been employed as a microbial fertilizer to increase pear production.

### General genome features

**Fig. 2:**
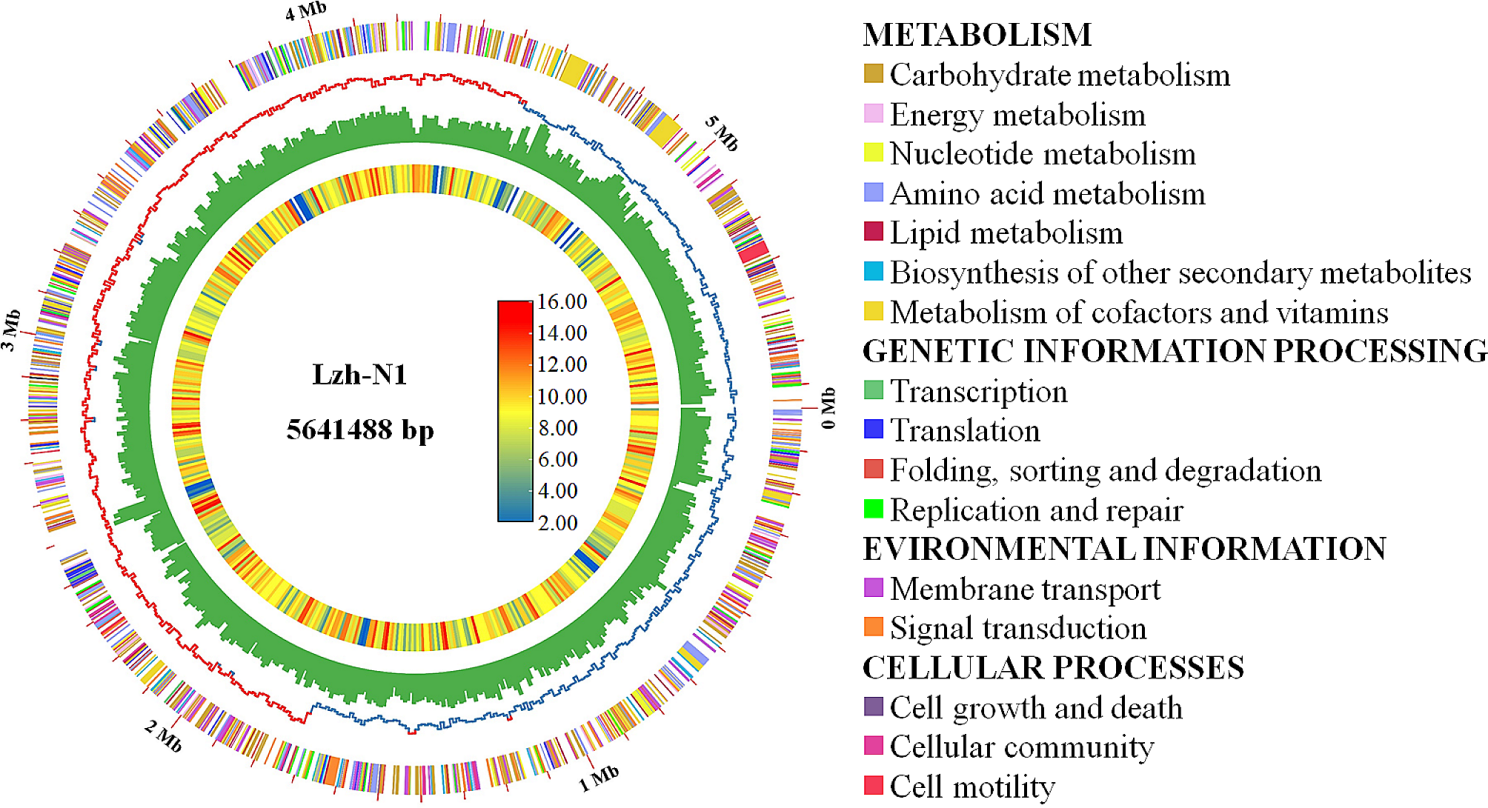
Circular genome map of *Paenibacillus* sp. lzh-N1. From the outside to the center, circle 1: the size of complete genome; circle 2: CDS predicted by KEGG, different colors represent different function classifications; circle 3: GC skew, with G% > C% in blue and G% < C% in red; circle 4: GC content; circle 5: the heatmap of gene density. The bar is for the heatmap

The assembled genome of *Paenibacillus* sp. lzh-N1 was found to be comprised of a single circular chromosome of 5,641,488 bp in length with a GC content of 45.50%, including 5,018 genes, 4,860 protein-coding genes, 109 tRNA, 45 rRNA, and 4 ncRNA (Table 1; Fig. 2). The genome data have been deposited in GenBank under the accession number of CP025696.1.

**Table 1:** The general genome feature of *Paenibacillus* sp. lzh-N1

Feature	Value
Genome size (bp)	5,641,488
G + C content (%)	45.5
Total number of genes	5,018
Total number of CDS	4,860
Protein-coding genes	4,656
Pseudo genes	204
tRNA	109
rRNA	45
ncRNA	4

**Fig. 3:**
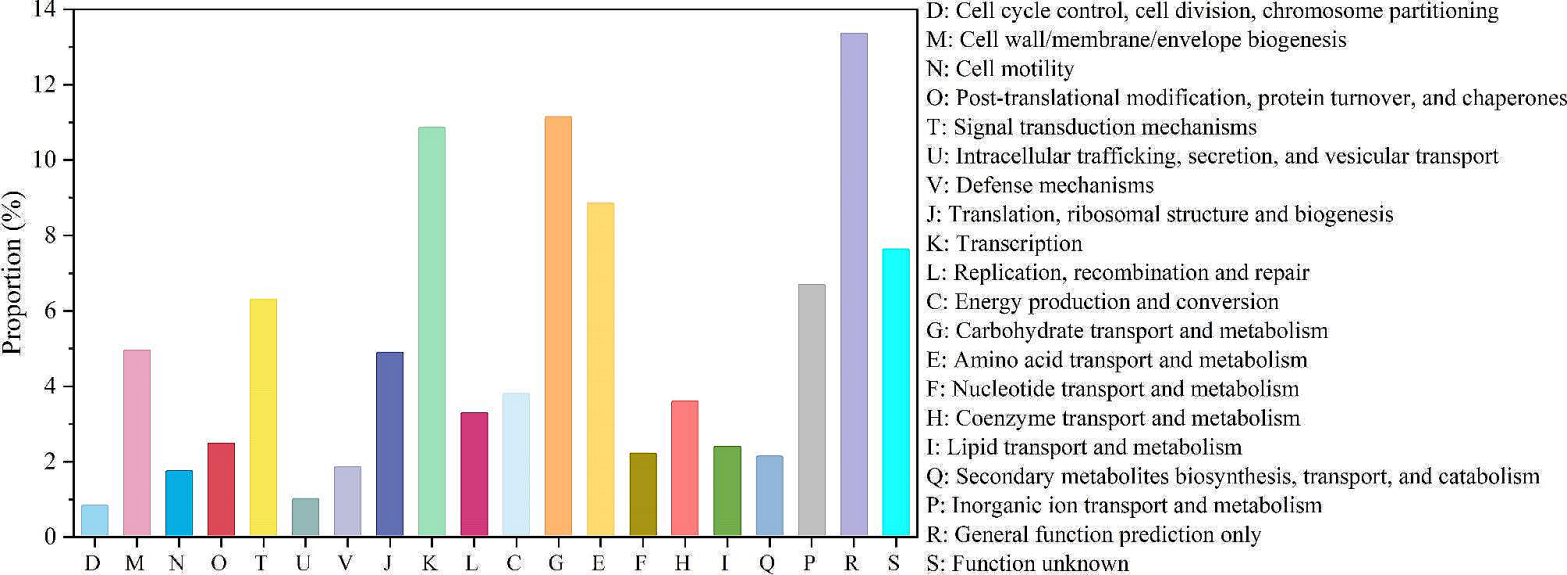
COG categories of *Paenibacillus* sp. lzh-N1

The protein-coding genes of *Paenibacillus* sp. lzh-N1 were classified into 20 different functional categories of Cluster of Orthologous Groups of proteins (COG, Fig. 3). The top three categories enriched by the genes included transcription (11.9%), carbohydrate transport and metabolism (11.1%), and amino acid transport and metabolism (8.8%). However, about 13.4% of the protein-coding genes were only function predicted, and about 7.6% were still poorly characterized.

### Phylogenetic analyses

**Fig. 4:**
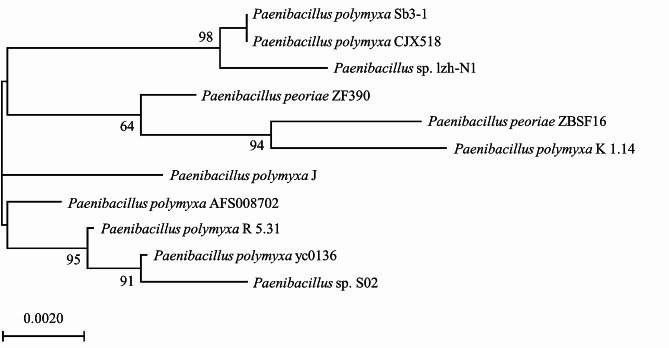
Phylogenetic tree of *Paenibacillus* sp. lzh-N1 based on 16 S rDNA sequences. The numbers in each branch points denote the percentages supported by bootstrap; Bar = 0.2% nucleotide divergence

**Fig. 5:**
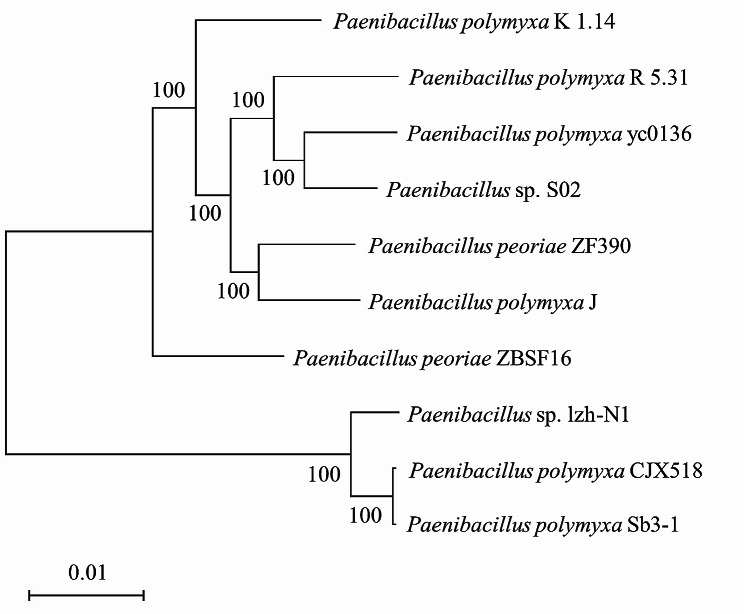
Phylogenetic tree of *Paenibacillus* sp. lzh-N1 based on whole genome sequences. The numbers in each branch points denote the percentages supported by bootstrap; Bar = 1% nucleotide divergence

Full-length 16 S ribosomal RNA gene (16 S rDNA) sequences (1425 nt) of *Paenibacillus* sp. lzh-N1 were obtained and deposited in GenBank under the accession number of KX865137.1. The gene sequence comparison with sequences in the GenBank database indicated that the strain lzh-N1 belonged to the genus *Paenibacillus*. The phylogenetic analysis of *Paenibacillus* sp. lzh-N1 with related *Paenibacillus* species based on 16 S rDNA sequences is shown in Fig. 4. *Paenibacillus* sp. lzh-N1 was clustered to *P. polymyxa*, and the closest relatives of the strain were *P. polymyxa* Sb3-1 and *P. polymyxa* CJX518. The phylogenetic analysis based on whole genome sequences is displayed in Fig. 5. Consistent with the results of phylogenetic analysis based on 16 S rDNA sequences, *Paenibacillus* sp. lzh-N1 was closest to *P. polymyxa* Sb3-1 and *P. polymyxa* CJX518.

### Average nucleotide identity (ANI) and genome-to-genome distance calculation (GGDC) analyses

**Fig. 6:**
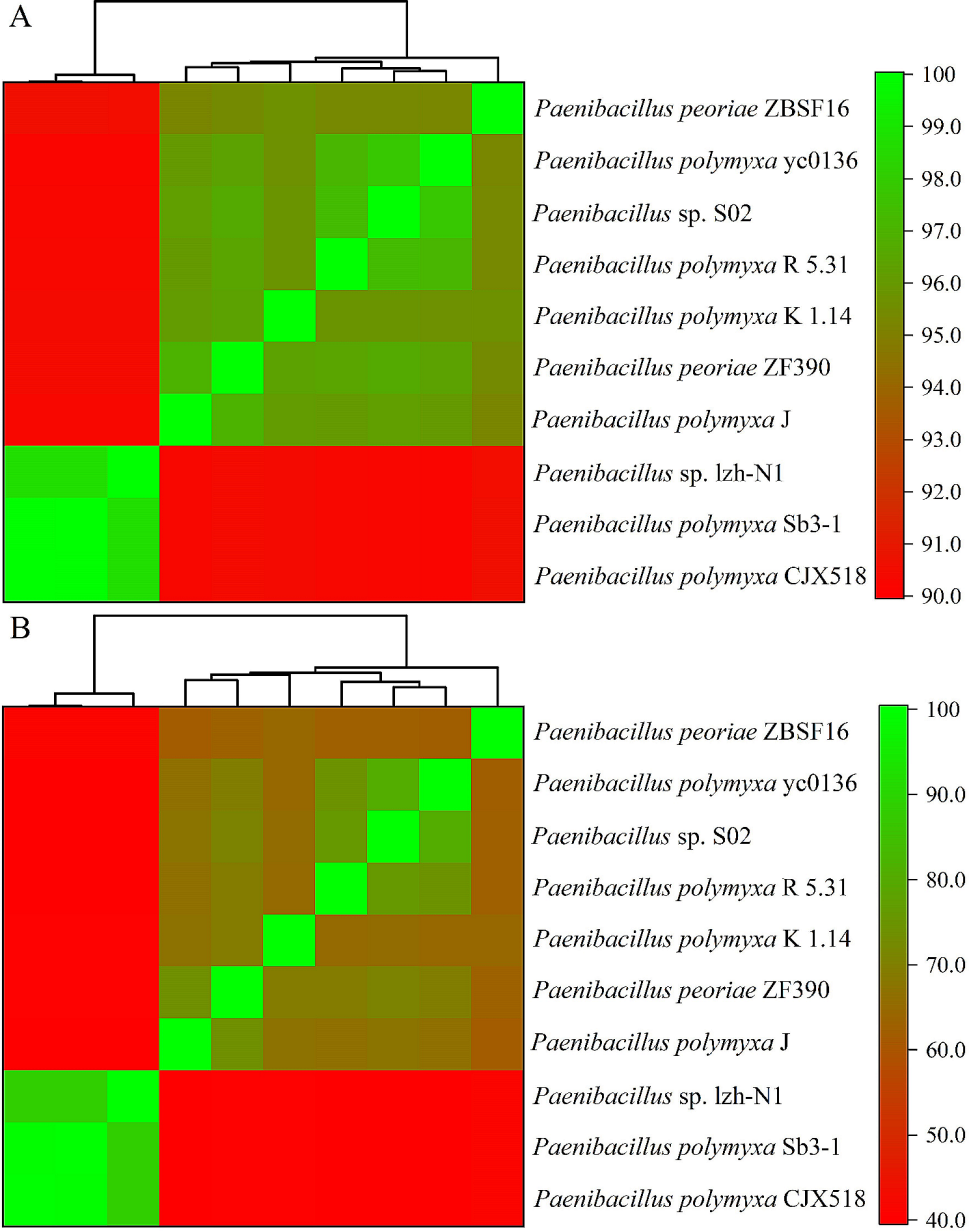
Heatmap analyses of ANI (**A**) and DDH (**B**) values. The color represents the ANI or DDH values. The green indicates the highest ANI or DDH value, and the red indicates the least ANI or DDH value

Two independent “digital DNA-DNA hybridization (dDDH)” methods, ANI and GGDC, were used to estimate the overall similarity between the genomes of two strains. The heatmap analysis of ANI values is shown in Fig. 6A. Genome comparison between *Paenibacillus* sp. lzh-N1 and *P. polymyxa* Sb3-1 and between *Paenibacillus* sp. lzh-N1 and *P. polymyxa* CJX518 generated an ANI value of 98.75% and 98.76%, respectively. It is higher than the 95% threshold for same species designation. The ANI values between *Paenibacillus* sp. lzh-N1 and other strains were less than 91%. The heatmap analysis based on the predicted DDH values is presented in Fig. 6B. For *Paenibacillus* sp. lzh-N1 and *P. polymyxa* Sb3-1 and for *Paenibacillus* sp. lzh-N1 and *P. polymyxa* CJX518, the predicted DDH values were 88.9% and 89.0%, respectively, much higher than the recommended 70% species-delimiting DDH value. The DDH values between *Paenibacillus* sp. lzh-N1 and other strains were less than 42%. The values of ANI and DDH are consistent, and it can be stated that *Paenibacillus* sp. lzh-N1 belongs to the same species as *P. polymyxa* Sb3-1 and *P. polymyxa* CJX518.

### Genetic foundation for the biosynthesis of antimicrobial metabolites

*Paenibacillus* sp. lzh-N1 exhibited significant antagonistic activity against *M. sentina* (Fr) Schrorter, indicating the existence of antimicrobial gene clusters. Sixteen secondary metabolic BGCs were identified by antiSMASH, including ribosomally synthesized and post-translationally-modified peptides (RiPPs), non-ribosomally produced peptides (NRPs), polyketides (PKs), NRPs-PKs hybrids, and cyclic lactone autoinducer (Table 2). Among these secondary metabolites, paenicidin [7], polymyxin B [8], aurantinin B [9], brevicidine [10], paenilipoheptin, and tridecaptin [11] have been proven to be active against bacteria, while fusaricidin B [8] and paenilarvins [12] have excellent antifungal activities. However, the functions of another four potential secondary metabolic BGCs are still unknown. These RiPP and betalactone metabolites may be related to the antimicrobial activity of the strain. Apart from these antimicrobial peptides, hydrolytic enzyme glucanase which can degrade the cell walls of various phytopathogens has antifungal effect [4]. Six genes encoding glucanase were found in the genome, and their locus_tags were C0638_03115, C0638_11415, C0638_14795, C0638_14995, C0638_20700, and C0638_20790, respectively. These findings provide a genetic basis for the antagonistic activity of *Paenibacillus* sp. lzh-N1.

**Table 2:** The potential gene clusters encoding secondary metabolites in *Paenibacillus* sp. lzh-N1

Type	Start	End	Similar cluster	Similarity
Lassopeptide	353,994	378,057	Paenicidin	40%
Proteusin	491,398	511,634	Unknown	
Transatpks-Nrps	617,929	692,636	Paenilarvins	50%
Nrps	1,585,487	1,648,051	Fusaricidin B	100%
Ranthipeptide	1,936,642	1,956,813	Unknown	
Nrps	2,467,859	2,548,757	Polymyxin B	100%
Ranthipeptide	3,034,140	3,059,526	Unknown	
Nrps-Transatpks-Otherks	3,724,696	3,825,086	Aurantinin B	35%
T1pks-Nrps	4,291,685	4,367,314	Brevicidine	60%
Betalactone	4,430,141	4,481,443	Unknown	
Transatpks-Nrps	4,551,237	4,678,191	Paenilipoheptin	30%
Nrps	4,849,526	4,943,336	Tridecaptin	100%
Cyclic lactone autoinducer, Nrps-like	5,316,759	5,378,445		
Lanthipeptide	5,519,292	5,545,741	Paenicidin B	71%
Cyclic lactone autoinducer	5,626,692	5,641,488		

### Regulation of biofilm formation

It has been reported that biofilm formation can enhance the antagonistic efficacy of many biocontrol agents, including *P. polymyxa* [13] and *Bacillus subtilis* [14]. Numerous studies have demonstrated that biofilm formation is regulated by QS systems in various bacteria [15]. Additionally, QS can regulate the production of some secondary metabolites in a cell density-dependent manner [16]. Therefore, QS may be important for the biocontrol activity of *Paenibacillus* sp. lzh-N1. There were two potential gene clusters producing cyclic lactone autoinducer (Table 2). Genes encoding the accessory regulator AgrB were found in two clusters with the locus_tags of C0638_23545 and C0638_25055, respectively. Furthermore, gene encoding S-ribosylhomocysteine lyase (LuxS, AUO05948.1) was also found in the genome. These results suggest that there are one or two QS systems in the strain. However, further studies are needed to clarify the roles of QS systems in regulating the biofilm formation and antagonistic activity of *Paenibacillus* sp. lzh-N1.

Exopolysaccharide (EPS) is an important component of the biofilms, which can influence the attachment and colonization of microbes to roots and root appendages [4]. Three genes were found to encode the EPS biosynthesis protein in the genome. The locus_tags of these genes were C0638_00325, C0638_11875, and C0638_20035, respectively. However, their functions in biofilm formation still await further investigations.

## Discussion

The pear leaf spot caused by *M. sentina* (Fr) Schrorter has received worldwide attention. If the attack of the fungus is intense, it will lead to premature defoliation and consequently yield losses. In this study, a strain, *Paenibacillus* sp. lzh-N1, was isolated from the rhizosphere soil of pear trees. The results of antagonistic experiments showed that *Paenibacillus* sp. lzh-N1 could significantly inhibit the growth of the pathogenic fungus. The results of species identification show that the strain belongs to the same species as *P. polymyxa* Sb3-1 and *P. polymyxa* CJX518. The biocontrol properties of *Paenibacillus* sp. lzh-N1 revealed its importance as a PGPR, which could provide great potential for agricultural applications.

The complete genome sequence of the strain was obtained and annotated to reveal the genetic foundation of its antagonistic ability. Fourteen antimicrobial peptides related to BGCs were successfully identified. The peptides included RiPPs, NRPs, PKs, and the NRPs-PKs hybrid. Among these peptides, fusaricidins and paenilarvins have outstanding antifungal activities [8, 12], which might be responsible for the antagonistic effects of *Paenibacillus* sp. lzh-N1 against the pathogenic fungus. In addition, several other peptides such as paenicidin, polymyxin, aurantinin, brevicidine, paenilipoheptin, and tridecaptin exhibit great antibacterial activities [7–11]. Therefore, it is speculated that *Paenibacillus* sp. lzh-N1 may have inhibitory activities against a wide range of microorganisms, making this strain a promising biocontrol agent. Besides, six genes encoding glucanase were found in the genome of *Paenibacillus* sp. lzh-N1. Hydrolytic enzymes (e.g., glucanase and chitinase) can degrade the cell walls of many phytopathogens [4]. Overexpression of glucanase from *P. polymyxa* A21 in *Streptomyces lydicus* has been reported to improve its biocontrol effect against the pathogenic fungus *Botrytis cinerea* [17]. Glucanase may also participate in the antifungal effects of *Paenibacillus* sp. lzh-N1.

Biofilm formation plays an important role in the attachment and colonization of the bacteria to the roots and can promote the antagonistic efficacy of biocontrol agents [4]. Generally, biofilm formation is strictly regulated by bacteria and affected by many environmental factors. QS is one of the crucial factors that regulate the formation of biofilm in various bacteria. Bacteria can monitor population density and regulate gene expression through QS [15]. There are two typical QS systems in Gram-positive bacteria. One QS system uses the autoinducing peptides (AIP) as signal molecules, and cyclic lactone-peptides are one group of AIP [18]. Two potential BGCs for the cyclic lactone autoinducer were found in the genome, suggesting that the strain contains this type of QS system. The other QS system uses autoinducer-2 (AI-2) as a signal molecule. The biosynthesis of AI-2 depends mainly on the expression of LuxS, which is widespread among both Gram-positive and Gram-negative bacteria [19]. Therefore, LuxS is the core of the AI-2-mediated QS system. In this study, a gene encoding LuxS was found in the genome. This indicates that the AI-2 mediated QS system exists in *Paenibacillus* sp. lzh-N1. The exact roles of QS systems in biofilm formation and whether QS can regulate the biosynthesis of antimicrobial peptides will be further elucidated.

## Conclusions

In the present study, a strain, *Paenibacillus* sp. lzh-N1, that could prevent pear leaf spots was isolated from the rhizosphere soil of pear trees. Analysis of its genome revealed the genetic foundation of its biocontrol properties, that is, antifungal peptides and glucanase might be responsible for the antagonistic effects of this strain. Moreover, potential QS systems were found in the bacterium, which might influence the biocontrol activity of *Paenibacillus* sp. lzh-N1 directly or indirectly. The potential antifungal molecules identified in this study lay a foundation for further studies on the genetic and biochemical pathways of *Paenibacillus* sp. lzh-N1 to improve its antagonistic efficiency and commercial application.

## Materials and methods

### Strain isolation

*Paenibacillus* sp. lzh-N1 was isolated from the rhizosphere soil samples of pear trees with brown spots in Taian, Shandong Province of China. This strain was chosen due to its antagonistic effects against the pathogen. The rhizosphere soil samples were collected and delivered to the laboratory at low temperature. The samples were diluted with sterile H_2_O and spread on the plates of LB and Gorodkowa′s medium. Colonies were selected after 24 h of incubation, and then inoculated on the plate of *M. sentina* (Fr) Schrorter. The colonies that could inhibit the growth of the pathogen were screened.

### Evaluation of antagonistic ability

The antagonistic ability of *Paenibacillus* sp. lzh-N1 was tested against *M. sentina* (Fr) Schrorter as follows. *M. sentina* (Fr) Schrorter was incubated on a plate of PDA medium at 30℃ for 48 h. Fresh colonies of *Paenibacillus* sp. lzh-N1 were inoculated on the sides of *M. sentina* (Fr) Schrorter and incubated at 30℃ for another 48–72 h.

### Genome sequencing, assembly and annotation

The genomic DNA of *Paenibacillus* sp. lzh-N1 was extracted and sequenced using the PacBio Sequel system. Raw data were filtered and assembled using SPAdes software (version 3.9.0(10)) to generate 1,307 Mb of total clean data, with a genome coverage of 232.0 ×. Gene annotation was performed using the NCBI Prokaryotic Genomes Automatic Annotation Pipeline. The function of each gene was further analyzed using four databases (Kyoto Encyclopedia of Genes and Genomes, KEGG; Gene Ontology, GO; COG; and Swiss-Prot). tRNA, rRNA, and sRNA were analyzed using the tRNAscan-SE 1.3.1, rRNAmmer 1.2, and Rfam, respectively. The circular genome map was constructed by TBtools [20], including general genome features and gene function annotation analyzed by KEGG [21–23]. The COG distribution was conducted by the Bacterial Pan Genome Analysis tool (BPGA) [24]. The potential BGCs were predicted with antiSMASH version 6.1.1 (http://antismash.secondarymetabolites.org/) [25].

### Phylogenetic analyses

Total genomic DNA for the analysis of 16 S rDNA sequence was isolated using the Bacterial Genomic DNA Isolation Kit (Tiangen, China) according to the manufactuter’s instructions. The 16 S rDNA sequence was amplified using the universal primers 27 F and 1492R as described previously [26]. The purified amplicons were sequenced by Sangon Biotech (Shanghai, China). The obtained 16 S rDNA sequence was compared with those available from the GenBank database (www.ncbi.nlm.nih.gov/genbank/). The phylogenetic trees based on 16 S rDNA and entire genome sequences were constructed with some species of the genus *Paenibacillus* by the approximately-maximum-likelihood method using FastTree software [27]. Whole genome sequences of the strains were downloaded from the GenBank database (https://www.ncbi.nlm.nih.gov/genome), and the GenBank accession numbers for these 16 S rDNA and genome sequences are listed in Table 3.

**Table 3:** The GenBank accession numbers of 16 S rDNA and genome sequences for *Paenibacillus* spp

Strain	GenBank accession numbers or locus_tag	
	16 S rDNA	Genome
*Paenibacillus* sp. lzh-N1	KX865137.1	CP025696.1
*Paenibacillus polymyxa* Sb3-1	RE92_16180	CP010268.1
*Paenibacillus polymyxa* CJX518	KF991241.1	CP029370.1
*Paenibacillus* sp. S02	LC385711.1	CP073682.1
*Paenibacillus polymyxa* AFS008702	OP986554.1	none
*Paenibacillus polymyxa* J	KT783525.1	CP015423.1
*Paenibacillus polymyxa* R 5.31	MN220144.1	CP097767.2
*Paenibacillus polymyxa* yc0136	EU430119.1	CP017967.3
*Paenibacillus polymyxa* K 1.14	MF622_000143	CP097778.3
*Paenibacillus peoriae* ZF390	IAQ67_00420	CP061172.1
*Paenibacillus peoriae* ZBSF16	MLD56_01685	CP092831.1

### ANI and GGDC analyses

ANI analysis was performed using the online tool provided by Majorbio (Shanghai, China; https://cloud.majorbio.com/page/tools/) [28]. GGDC analysis was performed using the program GGDC 3.0 provided by German Collection of Microorganisms and Cell Cultures (DSMZ; Braunschweig, Germany; http://ggdc.dsmz.de) [29]. The heatmaps based on the corresponding values of ANI and DDH were drawn using Origin 2018 software (OriginLab; Northampton, USA) [30].

## Data Availability

The 16 S rRNA sequence is available in GenBank with the accession number of KX865137.1 (https://www.ncbi.nlm.nih.gov/nuccore/KX865137.1), and the genome data are available in GenBank under the accession number of CP025696.1 (https://www.ncbi.nlm.nih.gov/nuccore/NZ_CP025696.1).
